# Childhood food insecurity trajectories and adolescent eating disorder symptoms: a UK cohort study

**DOI:** 10.1007/s00127-025-03022-y

**Published:** 2025-12-05

**Authors:** Nora Trompeter, Marie-Christine Opitz, Francisco Diego Rabelo-da-Ponte, Helen Sharpe, Sylvane Desrivieres, Ulrike Schmidt, Nadia Micali

**Affiliations:** 1 Great Ormond Street Institute of Child Health, UCL, 30 Guilford Street, London, WC1N 1EH UK; 2 Department of Clinical Psychology, School of Health in Social Sciences, University of Edinburgh, Edinburgh, UK; 3 Social Genetic and Developmental Psychiatry Centre, Institute of Psychiatry, Psychology and Neuroscience, King’s College London, London, UK; 4 Department of Psychological Medicine, Institute of Psychiatry, Psychology and Neuroscience, King’s College London, London, UK; 5 Center for Eating and feeding Disorders Research, Mental Health Center Ballerup, Copenhagen University Hospital – Mental Health Services CPH, Copenhagen, Denmark

**Keywords:** Food insecurity, Eating disorders, Disordered eating, Adolescence, Cohort study, ALSPAC

## Abstract

**Purpose:**

Food insecurity is increasingly linked with disordered eating. However, studies have not yet explored impacts of childhood food insecurity on disordered eating in adolescence. This study examined the links between patterns of childhood food insecurity and adolescent disordered eating.

**Methods:**

Data were from the Avon Longitudinal Study of Parents and Children (ALSPAC) cohort in the UK (6,723 participants; 55.7% girls). Food insecurity was reported by mothers from pregnancy to age 7. Disordered eating was reported by adolescents at ages 14, 16, and 18.

**Results:**

Most participants experienced no food insecurity throughout childhood (*n* = 5,801, 77.9%), followed by low food insecurity (*n* = 416, 12.4%), time-limited food insecurity (*n* = 292, 6.0%), and persistent food insecurity (*n* = 214, 3.7%). At age 14 adolescents in the time-limited food insecurity group had higher odds of binge eating (OR = 1.63, *p* = .040), and those in the persistent food insecurity group had higher odds of compensatory behaviours compared those in the no food insecurity group (OR = 1.72, *p* = .025). No significant associations were observed with disordered eating at age 16. At age 18 adolescents in the time-limited food insecurity group compared to the no food insecurity group had higher odds of compensatory behaviours (OR = 1.68, *p* = .041).

**Conclusions:**

Findings showed that childhood food insecurity was linked with higher odds of disordered eating in adolescence. Interestingly, associations were observed for those experiencing either time-limited or persistent food insecurity, highlighting the potential impact of early childhood experiences.

**Supplementary Information:**

The online version contains supplementary material available at https://doi.org/10.1007/s00127-025-03022-y.

Food insecurity describes the limited or uncertain access to nutritious food to support a healthy lifestyle [1], and is considered a social determinant of health, with robust links found between food insecurity and both physical health and mental health conditions [2, 3]. In the United Kingdom, 17% of households were classed as ‘food insecure’ in 2023 [1]. Children growing up in food-insecure households appear to be at particular risk of negative consequences of food insecurity, likely due to their developmental needs [4, 5]. More recently, research has pointed towards a link between food insecurity and eating disorders. A systematic review showed that adults experiencing food insecurity were significantly more likely to report current eating disorder symptoms, including both binge eating and compensatory eating behaviours, compared to adults not experiencing food insecurity [6]. Similarly, meta-analytic estimates suggest that adults who experience food insecurity are more than twice as likely to meet criteria for binge eating disorder compared to adults not experiencing food insecurity [7]. However, research among adolescents has been more limited, and most studies to date have been based in the United States.

Of the studies of adolescent eating disorder symptoms, cross-sectional research found that food insecurity is related to concurrent compensatory behaviours [8–10], with some evidence linking food insecurity to concurrent binge eating [8]. In contrast, longitudinal studies provide a clearer link between food insecurity and subsequent binge eating. A recent study by Nagata et al. [11] found that household food insecurity in early adolescence predicted binge eating, including meeting criteria for binge eating disorder, two years later. Similarly, Hazzard et al. [12] found that food insecurity in adolescence was associated with greater binge eating five years later. Interestingly, the same study also found that food insecurity was associated only with concurrent, but not subsequent, compensatory behaviours. In contrast, Hooper et al. [13] found no significant associations between adolescent food insecurity and either binge eating or compensatory behaviours in early adulthood. Thus, there is mixed evidence on the long-term outcomes of food insecurity and eating disorder behaviours in adolescence.

Of note, all available studies with adolescents have focused on food insecurity during adolescence and have not explored childhood food insecurity. Early childhood experiences with food, including parental feeding practices, food preferences, and food availability have been shown to have long-term impacts on eating habits [14–16]. Thus, these early experiences may shape the relationships children form with food and eating, potentially contributing to disordered eating in adolescence. Further, studies have focused on single timepoints of food insecurity and have not considered the potential transient nature of food insecurity or effects of persistent food insecurity with limited longitudinal evidence available [10]. Such a distinction has important policy implications as it provides insights into how potential interventions should be allocated. Previous studies on food insecurity transitions during childhood indicate that both persistent and intermittent food insecurity are associated with mental health and cognitive skills in later childhood, suggesting that the adverse effects of food insecurity may last despite food becoming available [4, 17]. However, no such research has examined outcomes regarding eating disorder symptoms in adolescents. Varying levels of food insecurity may exacerbate the ‘feast or famine’ cycle hypothesized to explain the link between food insecurity and eating disorder symptoms [18]. Indeed, adults seeking treatment for binge eating have reported that past experiences of food insecurity contributed to their eating disorder symptoms due to both varying levels of food availability that shaped eating habits and associating availability of food with safety and comfort that shaped emotional eating habits [19].

Overall, while current research supports a relationship between food insecurity and increased eating disorder behaviours among adolescents, little is known about the relationship between childhood food insecurity and adolescent eating disorder symptoms. This is particularly important to understand, as early-life patterns of food insecurity may have lasting impacts on eating disorder symptoms that are not captured by research on concurrent food insecurity. To address these gaps, the current study examined the links between childhood food insecurity and adolescent eating disorder symptoms. It was hypothesised that food insecurity during childhood would be positively associated with binge eating, but not compensatory behaviours due to inconsistent findings in previous research.

## Methods

### Participants and procedure

The current study used data from the Avon Longitudinal Study of Parents and Children (ALSPAC) cohort in the UK. The ALSPAC Cohort [20–22] recruited pregnant women living in the geographical area of Avon, UK, who were expected to deliver between April 1, 1991 and December 31, 1992. In total, 14,541 pregnancies were enrolled and 13,988 children from these pregnancies were alive at 1 year. An additional 913 children were enrolled during later phases of enrollment at age 7. Participants varied in terms of socio-economic status, with a slightly higher socio-economic status among mothers in the ALSPAC study as compared to the UK as a whole, as indicated by higher proportions of home ownership and car ownership [22].Ethical approval was granted by the ALSPAC Law and Ethics Committee and Local Ethics Committees. Informed consent for the use of data collected via questionnaires and clinics was obtained from participants following the recommendations of the ALSPAC Ethics and Law Committee at the time. A fully searchable ALSPAC data dictionary is available at https://www.bristol.ac.uk/alspac/researchers/our-data.

For the current study, we used data from all participants that had information available on food insecurity for at least one timepoint during childhood, and had completed measures on eating disorder behaviours at least at one timepoint during adolescence. As such, only children from the original birth cohort and not those from later enrollment phases were included due to childhood food insecurity being measured prior to additional recruitment phases. Among child twin pairs, one twin per pair was randomly excluded from the current study. The final analytic sample included 6,723 adolescents (55.67% female, 44.33% male).

### Measures

#### Food insecurity

Food insecurity was measured using a single item (“*How difficult at the moment do you find it to afford these items: food*”) in mother-reported surveys at pregnancy, 8 months, 2 years, 3 years, 5 years, and 7 years. We coded responses as 0 (no; grouping “not difficult”, “slightly difficult”) or 1 (yes; grouping “very difficult,” “fairly difficult“). The measure has previously been used in ALSPAC to investigate financial difficulties [23]. Single-item measures of food insecurity have been shown to highly correlate with multi-item measures, but are more conservative in their prevalence estimates [24, 25]. Additionally, partners reported on food insecurity at ages 8 months, 2 years, 3 years, 5 years, and 7 years. For sensitivity analyses, we created a composite variable of food insecurity reported by either mother or partner. Partner reports were included only if partners were unchanged since enrolment to ensure continuity. Group-based trajectory modelling was used to create categories of food insecurity (see data analysis plan for further information). After estimating trajectory groups, each participant was assigned a probability of group membership and allocated to the group with the highest probability.

#### Disordered eating

Data on eating disorder behaviours at age 14, 16, and 18 were collected using questions adapted from the Youth Risk Behavior Surveillance System questionnaire [26]. The measure covered binge eating, fasting, dieting, and excessive exercise in the previous year. Binge eating was defined as eating a large amount of food and having a feeling of loss of control during that episode. Purging was defined as participants having used of laxatives or self-induced vomiting to lose weight or avoid gaining weight. Fasting was defined as not eating at all for at least a day, to lose weight or avoid gaining weight. Excessive exercise was defined having exercised for weight loss purposes, and either feeling guilty about missing exercise, continuing exercise when sick or injured or having difficulty meeting other obligations due to exercise. All variables were coded as binary yes/no responses. For the current study we used data on binge eating and a binary composite variable for any compensatory behaviours (i.e., any fasting, purging or excessive exercise in the last year).

#### Covariates

To control for potentially confounding variables, we also included measures on parental education (A levels or higher, lower than A levels) for both mothers and fathers, parental mental health at age 3 as measured by the Edinburgh Postnatal Depression Score [27] and Crown-Crisp Experiential Index [28], maternal ethnicity, child sex, child ethnicity and child BMI at age 9.5 based on objective measures of height and weight.

### Data analysis

All analyses were conducted in Stata version 18. First, we determined different food insecurity trajectories based on mother-reported food insecurity from pregnancy to age 7 using group-based trajectory modelling using the traj plugin [29]. Models of 2- to 6-trajectory groups were estimated using full information maximum likelihood based on the maximum available sample. A final model was selected based on their Bayesian Information Criterion (BIC), Akaike Information Criterion (AIC), entropy values, an adequate sample size in each group (i.e., >50 participants per group), and the interpretability of the model. For the main analyses, we examined associations between trajectories of food insecurity and eating pathology outcomes using binary regression analyses. In total, six separate regression analyses were conducted for two different outcome variables (binge eating and compensatory behaviours) at three timepoints (age 14, 16, and 18). All analyses controlled for covariates.

Similar to other cohort studies, ALSPAC suffers from both attrition and sporadic non-responding which are linked primarily to greater social disadvantage [30]. To mitigate potential biases, we controlled for socio-economic factors at baseline and included these in imputation methods. Missing data on covariates was imputed using multiple imputations with chained equations (MICE) with 30 imputations. All predictor and outcome variables were used in the imputation model, as well as BMI based on mother-report, maternal ethnicity and paternal ethnicity. Continuous variables were imputed using regression equations and limited in range to that of the observed values, binary variables were imputed using logistic regression equations. Imputation was performed on the whole sample, prior to individual analyses. Missing data on outcome variables was not imputed. High levels of missing data were present across control variables, especially on paternal mental health (< 40%). As such, imputed datasets were evaluated on fraction of missing information (FMI) to determine appropriateness of the models. FMI describes the proportion of the total sampling variance due to missing data and is seen as a better indicator than amount of missing data [31]. Largest FMI on the imputed models ranged between 0.28 and 0.42. Full model statistics are reported in Supplement (1) Additional sensitivity analyses were conducted with minimally adjusted (not controlling for BMI) and unadjusted results presented in Supplementary (2) Patterns of findings were similar to those in the fully-adjusted analyses, which are presented below.

## Results

### Food insecurity trajectories

In the overall sample, 391 (6.1%) participants were classified as food insecure during pregnancy, 530 (8.3%) participants were classified as food insecure at 8-months of age, 477 (7.7%) participants were classified as food insecure at age 2, 463 (7.7%) participants were classified as food insecure at age 3, 274 (4.7%) participants were classified as food insecure at age 5, and 180 (3.2%) participants were classified as food insecure at age 7. To determine trajectories of food insecurity, we fitted logistic group-based trajectory models for 2–6 groups allowing for liner, quadratic and cubic trends. The 4-group solution exhibited the best fit and interpretability (full model statistics are reported in Supplementary 2). Most participants were classified as having no food insecurity throughout childhood (*n* = 5,801, 77.93%), followed by low food insecurity (*n* = 416, 12.37%), time-limited food insecurity (*n* = 292, 6.01%), and persistent food insecurity (*n* = 214, 3.69%).

**Fig. 1:**
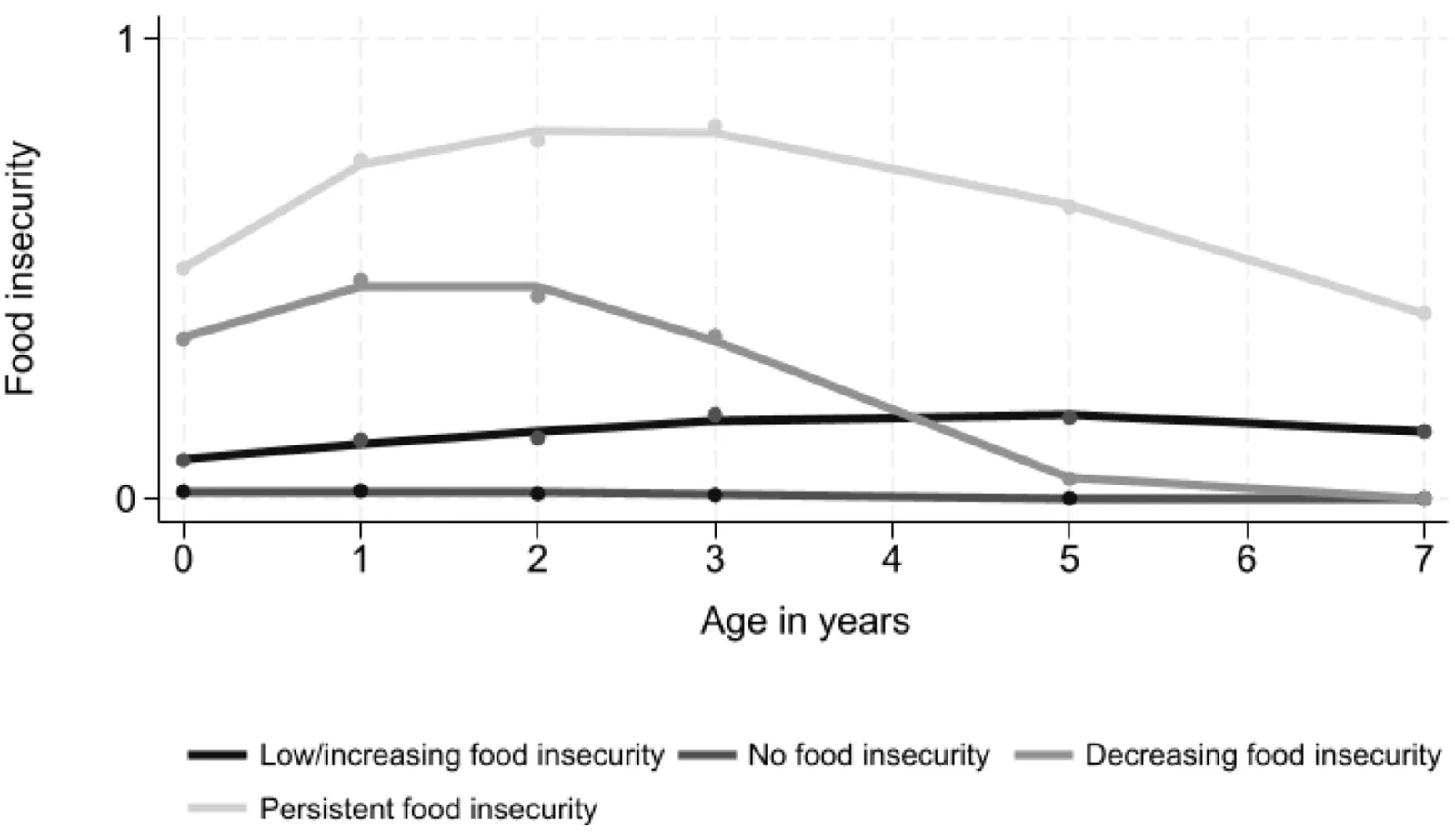
Food insecurity trajectories based on mother-report

Groups differed significantly in child BMI (*F*(3,5289) = 7.23, *p* < .001), maternal education (*X*(3) = 72.47, *p* < .001, *V* = 0.11), paternal education (*X*(3) = 72.51, *p* < .001, *V* = 0.12), maternal depression(*F*(3,5951) = 98.00, *p* < .001), paternal depression(*F*(3,3744) = 8.47, *p* < .001), maternal anxiety (*F*(3,5957) = 79.15, *p* < .001), and paternal anxiety (*F*(3,3728) = 3.37, *p* = .018). No differences were observed in child sex (*X*(3) = 3.43, *p* = .330, *V* = 0.02), child ethnicity (*X*(3) = 7.38, *p* = .061, *V* = 0.04), maternal ethnicity (*X*(3) = 2.48, *p* = .479, *V* = 0.02), or paternal ethnicity (*X*(3) = 1.95, *p* = .583, *V* = 0.02). See Table 1 for further detail.

**Table 1:** Sample characteristics by food insecurity trajectory group. N (%)

	No food insecurity	Low food insecurity	Time-limited food insecurity	Persistent food insecurity	Total
N	5,801	416	292	214	6,723
Child sex					
Female	3,224 (55.6)	222 (53.4)	167 (57.2)	130 (60.8)	3,743 (55.7)
Male	2,577 (44.4)	194 (46.6)	125 (42.8)	84 (39.2)	2,980 (44.3)
Child ethnicity					
White	4,635 (96.7)	319 (96.1)	208 (93.7)	145–150 (~ 95.0)	5,308 (96.5)
Black or minority ethnicity	157 (3.3)	10–15 (~ 4.0)	10–15 (~ 6.0)	NR	192 (3.5)
Child BMI at age 9 (M, SD)	17.6 (2.8)	17.7 (2.8)	18.3 (3.2)	18.3 (3.5)	17.6 (2.8)
Maternal education					
A-levels or higher	2,068 (41.9)	102 (28.9)	55 (23.8)	39 (22.5)	2,264 (39.7)
Lower than A-levels	2,872 (58.1)	251 (71.1)	176 (76.2)	134 (77.5)	3,433 (60.3)
Paternal education					
A-levels or higher	2,684 (59.1)	135 (43.1)	80 (42.6)	44 (35.5)	2,943 (57.0)
Lower than A-levels	1,854 (40.9)	178 (56.9)	108 (57.4)	80 (64.5)	2,220 (43.0)
Maternal ethnicity					
White	5,518 (98.0)	390–395 (~ 98.0)	275–280 (~ 96.0)	195–200 (~ 97.0)	6,385 (97.9)
Black or minority ethnicity	114 (2.0)	NR	NR	NR	137 (2.1)
Paternal ethnicity					
White	4,568 (97.7)	313 (96.9)	200–205 (~ 98.0)	130–135 (~ 98.0)	5,213 (97.8)
Black or minority ethnicity	105 (2.3)	10 (3.1)	NR	NR	120 (2.3)
Maternal depression (M, SD)	5.6 (4.6)	7.8 (5.6)	8.3 (5.3)	10.4 (6.1)	6.0 (4.9)
Paternal depression (M, SD)	3.6 (3.7)	4.0 (4.4)	4.6 (4.1)	5.4 (4.1)	3.7 (3.8)
Maternal anxiety (M, SD)	4.3 (3.3)	5.8 (4.1)	5.9 (3.8)	7.3 (4.2)	4.6 (3.4)
Paternal anxiety (M, SD)	3.1 (2.6)	3.4 (3.2)	3.6 (3.1)	3.7 (2.5)	3.1 (2.7)

Note. M = mean score, SD = standard deviation. NR = Not reported due to low frequencies. Ranges are reported for values where ‘NR’ values could otherwise be inferred

### Disordered eating at age 14

At age 14, *n* = 5,122 individuals had data available on binge eating and *n* = 5,759 individuals on compensatory behaviours. Of these, 326 (6.4%) reported engaging in any binge eating and 786 (13.7%) reported engaging in any compensatory behaviours in the previous year.

At age 14, belonging to the time-limited food insecurity group was associated with higher odds of binge eating compared to the no food insecurity group (OR = 1.63 [1.02; 2.61], *p* = .040). No significant differences were found between belonging to the other food insecurity trajectories compared to the no food insecurity trajectory (low food insecurity: OR = 0.97 [0.59; 1.57], *p* = .895; persistent food insecurity OR = 0.91 [0.49; 1.69], *p* = .760). Regarding compensatory behaviours, belonging to the persistent food insecurity group was associated with higher odds of compensatory behaviours compared to the no food insecurity group (OR = 1.72 [1.07; 2.78], *p* = .025). No significant differences were found between belonging to the other food insecurity trajectories compared to the no food insecurity trajectory (low food insecurity: OR = 0.97 [0.66; 1.43], *p* = .896; time-limited food insecurity OR = 1.35 [0.88; 2.06], *p* = .166). No other group comparisons were significant.

## Disordered eating at age 16

At age 16, *n* = 4,089 individuals had data available on binge eating and *n* = 4,769 individuals on compensatory behaviours. Of these, 521 (12.7%) reported engaging in any binge eating and 1,326 (27.8%) reported engaging in any compensatory behaviours in the previous year.

No significant associations were found between belonging to any of the food insecurity trajectories compared to the no food insecurity trajectory and binge eating (low food insecurity: OR = 1.25 [0.80; 1.96], *p* = .323; time-limited food insecurity: OR = 1.66 [0.96; 2.89], *p* = .071; persistent food insecurity OR = 1.40 [0.71; 2.76], *p* = .333). Similarly, no significant associations were found between belonging to any of the food insecurity trajectories compared to the no food insecurity trajectory and compensatory behaviours (low food insecurity: OR = 0.94 [0.67; 1.32], *p* = .717; time-limited food insecurity: OR = 0.92 [0.59; 1.43], *p* = .706; persistent food insecurity OR = 1.37 [0.83; 2.27], *p* = .224). No other group comparisons were significant. Of note, the odds ratios and confidence intervals for the persistent food insecurity comparisons were large, indicating issues with statistical power.

## Disordered eating at age 18

At age 18, *n* = 2,324 individuals had data available on binge eating and *n* = 3,164 individuals on compensatory behaviours. Of these, 431 (18.5%) reported engaging in any binge eating and 845 (26.7%) reported engaging in any compensatory behaviours in the previous year.

No significant associations were found between belonging to any of the food insecurity trajectories compared to the no food insecurity trajectory and binge eating (low food insecurity: OR = 0.99 [0.57; 1.74], *p* = .983; time-limited food insecurity: OR = 0.94 [0.47; 1.88], *p* = .862; persistent food insecurity OR = 1.84 [0.90; 3.74], *p* = .092). Belonging to the time-limited food insecurity compared to the no food insecurity trajectory was associated with higher odds of compensatory behaviours (OR = 1.68 [1.02; 2.75], *p* = .041). No differences were found with the low food insecurity (OR = 1.46 [0.98; 2.18], *p* = .062) or persistent food insecurity trajectory (OR = 1.74 [0.96; 3.18], *p* = .069). No other group comparisons were significant. Of note, the odds ratios and confidence intervals for the persistent food insecurity comparisons were large, indicating issues with statistical power.

## Discussion

In this UK population-based cohort study, we found four distinct trajectories of food insecurity throughout childhood. While most children consistently experienced no food insecurity, about a quarter of participants experienced some level of food insecurity throughout childhood. These food insecurity trajectories were broadly classed as no food insecurity, low food insecurity, time-limited food insecurity and persistent food insecurity. Belonging to the persistent food insecurity trajectory was associated with higher odds of engaging in compensatory behaviours at age 14, whereas belonging to the time-limited food insecurity trajectory was associated with higher odds of engaging in binge eating at age 14 and compensatory behaviours at age 18, compared to the no food insecurity trajectory. Additionally, effect sizes pointed to a potential association between persistent food insecurity and both binge eating and compensatory behaviours at age 18, however, this association did not reach significance. No differences were found for those belonging to the low food insecurity trajectory. Results showed significant associations between food insecurity and both binge eating and compensatory behaviours. Interestingly, no significant associations were found at age 16. This may be due to limited statistical power, similar to age 18 outcomes, or due to disordered eating symptoms becoming more pronounced in later stages of development. Indeed, our findings showed an increase in disordered eating throughout adolescence, especially for binge eating. This is in line with previous research suggesting that binge eating tends to develop later than compensatory behaviours [32].

Findings point to several potential underlying mechanisms, on one hand engaging in binge eating might serve as a potential mediator linking food insecurity with compensatory behaviours, as those with time-limited food insecurity were significantly more likely to engage in binge eating, but not compensatory behaviours, at age 14, and more likely to engage in compensatory behaviours, but not binge eating, at age 18, compared to those with no food insecurity. For instance, belonging to the persistent food insecurity trajectory was associated with higher odds of engaging in compensatory behaviours at age 14. On the other hand, persistent food insecurity may trigger earlier disordered eating patterns, resulting in early compensatory behaviours at age 14. However, further research is needed to explore such pathways. Relatedly, weight status may serve as a mediator whereby childhood food insecurity leads to increased body weight, which in turn places adolescents at greater risk for engaging in disordered eating behaviours, in particular compensatory behaviours [33]. Indeed, our findings showed that children’s BMI differed by trajectory group, with those in the persistent food insecurity group reporting the highest BMI by age 10. However, formal analysis of such mediating factors needs to be conducted to further understand the underlying mechanisms at play.

Findings from the current study support increasing evidence linking food insecurity to eating disorder symptoms [6]. Importantly, these findings contradict the narrative that eating disorders are primarily disorders of ‘affluence’ [34]. Indeed, findings show that eating disorders not only occur across the socio-economic spectrum, but that children growing up in food insecurity are more likely to report eating disorder symptoms compared to those who never experience food insecurity.

Interestingly, both persistent and time-limited food insecurity were linked with eating disorder symptoms in the current study. These findings suggest that the adverse effects of food insecurity may continue to impact children’s development even if food security is obtained, or that early childhood represents a specific window of vulnerability when food insecurity might be especially detrimental to long-term outcomes. Childhood food insecurity impacts not only children themselves, but their parents as well. Parents in food insecure households have been shown to engage in more restrictive feeding practices, have fewer family meals, and engage in disordered eating themselves [35, 36]. This has critical implications for public health policies. On one level it highlights the need for early intervention to reduce food insecurity as early as possible. Data from the current study shows that the time-limited food insecurity group saw changes in their trajectory around 5 years of age, when children likely started school. This may be explained by several factors, including provision of free school meals, reduced childcare costs, and increased time for parents in the workforce. Despite this, adolescents in the time-limited food insecurity trajectory had higher odds of engaging in eating disorder behaviours akin to those in the persistent food insecurity trajectory. On another level, these findings highlight the need to not only tackle food insecurity itself, but also the mechanisms linking food insecurity to eating disorder symptoms. For example, interventions that target both food insecurity and nutritional needs through family-meal education programs have shown success in reducing food insecurity and increasing family meals [37]. Research should consider adding elements of eating disorder prevention to such interventions to further increase their effectiveness.

While the current study is a critical starting point for understanding how childhood food insecurity is associated with adolescent eating disorder symptoms, it does not address potential mechanisms. Future research should test these mechanisms, including parenting practices and broader mental health impacts, to provide tangible targets to improve early intervention programs. Several additional limitations should be acknowledged. Importantly, the data on childhood food insecurity was collected during the 1990s in the UK and need to be replicated in with more recent data. Children in the current study faced a different social and economic climate compared to current generations with less policy focus on alleviating food insecurity and higher unemployment rates [38, 39]. For example, several effective initiatives targeting food insecurity such as food banks or school breakfast clubs were not established in the UK until the 2000s [38]. Relatedly, at the time of data collection no established measures of food insecurity in the UK existed and the measure in the current study relied on a single-item measure capturing financial difficulty obtaining food [40]. While this is a core component of food insecurity, other aspects such as food anxiety frequently captured in multi-item measures may be missed. The current study was further limited in statistical power for some trajectory groups. Thus, there may have been subtle differences between the persistent and time-limited food insecurity groups that we were not able to detect. Similarly, associations with outcomes at ages 16 and 18 were impacted by higher drop-out levels that likely reduced statistical power in these analyses. This is, in part, evident through the large confidence intervals seen in some analyses. Lastly, while we controlled for a range of co-occurring factors such as parental mental health and child BMI, it is important to acknowledge that food insecurity does not occur in isolation and has wide ranging impacts on both parents and children, which can exacerbate the development of eating disorder symptoms.

## Conclusion

The current study showed that childhood food insecurity was linked with higher odds of disordered eating at age 14 and 18, but not 16. Interestingly, associations were observed for those experiencing both time-limited and persistent food insecurity, highlighting the potential impact of early childhood experiences. Future research should consider identifying potential mechanisms to further inform potential early intervention programs.

## Supplementary Information

Below is the link to the electronic supplementary material.

Supplementary Material 1

Supplementary Material 2

Supplementary Material 3

## Data Availability

Access to ALSPAC data is through a system of managed open access ( [http://www.bristol.ac.uk/alspac/researchers/access/](http:/www.bristol.ac.uk/alspac/researchers/access) ). Code for the current paper is available through OSF (https://osf.io/x25jf/?view\_only=ef8d57a3711b472e9a2a06bb969baf57).
