# Supplementary material for: Childhood food insecurity trajectories and adolescent eating disorder symptoms: a UK cohort study
Source: Soc Psychiatry Psychiatr Epidemiol. 2025 Dec 5;61(10):2013–21. doi: 10.1007/s00127-025-03022-y (PMC13586018; doi:10.1007/s00127-025-03022-y)
Supplement: Supplementary file 1 [file 127_2025_3022_MOESM1_ESM.docx]

**Imputation model summary statistics**

*Binge eating at age 14:*

Multiple-imputation estimates Imputations = **30**

Logistic regression Number of obs = **5,122**

Average RVI = **0.1599**

Largest FMI = **0.4214**

DF adjustment: **Large sample** DF: min = **168.76**

avg = **253,308.57**

max = **2078052.72**

Model F test: **Equal FMI** F( **12**,**15705.6**) = **6.94**

Within VCE type: **OIM** Prob > F = **0.0000**

*Compensatory behaviours at age 14:*

Multiple-imputation estimates Imputations = **30**

Logistic regression Number of obs = **4,542**

Average RVI = **0.1262**

Largest FMI = **0.3437**

DF adjustment: **Large sample** DF: min = **253.05**

avg = **158,009.08**

max = **1629408.19**

Model F test: **Equal FMI** F( **14**,**28228.4**) = **9.47**

Within VCE type: **OIM** Prob > F = **0.0000**

*Binge eating at age 16:*

Multiple-imputation estimates Imputations = **30**

Logistic regression Number of obs = **3,254**

Average RVI = **0.1132**

Largest FMI = **0.2862**

DF adjustment: **Large sample** DF: min = **364.00**

avg = **393,511.17**

max = **4761569.98**

Model F test: **Equal FMI** F( **14**,**34253.2**) = **10.82**

Within VCE type: **OIM** Prob > F = **0.0000**

*Compensatory behaviours at age 16:*

Multiple-imputation estimates Imputations = **30**

Logistic regression Number of obs = **3,819**

Average RVI = **0.1058**

Largest FMI = **0.2905**

DF adjustment: **Large sample** DF: min = **353.39**

avg = **60,108.26**

max = **553,076.17**

Model F test: **Equal FMI** F( **14**,**38741.4**) = **27.21**

Within VCE type: **OIM** Prob > F = **0.0000**

*Binge eating at age 18*

Multiple-imputation estimates Imputations = **30**

Logistic regression Number of obs = **1,865**

Average RVI = **0.1089**

Largest FMI = **0.3671**

DF adjustment: **Large sample** DF: min = **222.00**

avg = **420,947.99**

max = **2600828.34**

Model F test: **Equal FMI** F( **14**,**36665.7**) = **5.00**

Within VCE type: **OIM** Prob > F = **0.0000**

*Compensatory behaviours at age 18:*

Multiple-imputation estimates Imputations = **30**

Logistic regression Number of obs = **2,557**

Average RVI = **0.1174**

Largest FMI = **0.3482**

DF adjustment: **Large sample** DF: min = **246.57**

avg = **156,019.68**

max = **1154887.85**

Model F test: **Equal FMI** F( **14**,**32104.7**) = **13.79**

Within VCE type: **OIM** Prob > F = **0.0000**
