# Supplementary material for: Childhood food insecurity trajectories and adolescent eating disorder symptoms: a UK cohort study
Source: Soc Psychiatry Psychiatr Epidemiol. 2025 Dec 5;61(10):2013–21. doi: 10.1007/s00127-025-03022-y (PMC13586018; doi:10.1007/s00127-025-03022-y)
Supplement: Supplementary file 2 [file 127_2025_3022_MOESM2_ESM.docx]

Supplementary materials

**Table S1.** Trajectory model fit statistics

|  | Mother-report | | | | Composite-report | | | |
| --- | --- | --- | --- | --- | --- | --- | --- | --- |
| Model | BIC | AIC | ll | Entropy | BIC | AIC | ll | Entropy |
| 2-classes | -7282.51 | -7255.25 | -7247.25 | .87 | -8419.80 | -8392.55 | -8384.55 | .89 |
| 3-classes | -7269.43 | -7228.55 | -7216.55 | .64 | -7936.80 | 7895.92 | -7883.92 | .76 |
| 4-classes | -7277.21 | -7219.30 | -7202.30 | .70 | -7917.32 | -7866.22 | -7851.22 | .61 |
| 5-classes | -7288.94 | -7220.81 | -7200.81 | .63 | -7911.87 | -7822.48 | -7801.48 | .70 |
| 6-classes | -7296.88 | -7215.13 | -7191.13 | .66 | -7944.26 | -7869.32 | -7847.32 | .52 |
