# Supplementary material for: Childhood food insecurity trajectories and adolescent eating disorder symptoms: a UK cohort study
Source: Soc Psychiatry Psychiatr Epidemiol. 2025 Dec 5;61(10):2013–21. doi: 10.1007/s00127-025-03022-y (PMC13586018; doi:10.1007/s00127-025-03022-y)
Supplement: Supplementary file 3 [file 127_2025_3022_MOESM3_ESM.docx]

Table SX. Minimally adjusted results (excluding BMI).

|  | Binge eating at 14 | Compensatory behaviours at 14 | Binge eating at 16 | Compensatory behaviours at 16 | Binge eating at 18 | Compensatory behaviours at 18 |
| --- | --- | --- | --- | --- | --- | --- |
| No food insecurity (reference) | - | - | - | - | - | - |
| Low food insecurity | 0.96 [0.60; 1.56] | 1.00 [0.72; 1.39] | 1.09 [0.73; 1.62] | 0.99 [0.74; 1.33] | 1.07 [0.66; 1.73] | 1.34 [0.94; 1.91] |
| Time-limited food insecurity | **1.72 [1.08; 2.73]** | **1.61 [1.15; 2.24]** | 1.46 [0.93; 2.29] | 0.96 [0.68; 1.35] | 1.18 [0.69; 2.01] | 1.48 [0.98; 2.23] |
| Persistent food insecurity | 0.96 [0.52; 1.79] | **1.58 [1.09; 2.28]** | 1.58 [0.94; 2.66] | 1.33 [0.89; 1.97] | 1.39 [0.74; 2.62] | 1.48 [0.90; 2.41] |

Table SX. Unadjusted results

|  | Binge eating at 14 | Compensatory behaviours at 14 | Binge eating at 16 | Compensatory behaviours at 16 | Binge eating at 18 | Compensatory behaviours at 18 |
| --- | --- | --- | --- | --- | --- | --- |
| No food insecurity (reference) | - | - | - | - | - | - |
| Low food insecurity | 1.05 [0.66; 1.68] | 1.04 [0.75; 1.42] | 1.16 [0.79; 1.71] | 0.99 [0.75; 1.31] | 1.23 [0.77; 1.96] | 1.32 [0.94; 1.85] |
| Time-limited food insecurity | **1.88 [1.20; 2.94]** | **1.68 [1.22; 2.32]** | 1.49 [0.97; 2.28] | 0.94 [0.68; 1.30] | 1.34 [0.80; 2.24] | 1.45 [0.98; 2.14] |
| Persistent food insecurity | 1.16 [0.64; 2.11] | **1.74 [1.22; 2.49]** | **1.81 [1.11; 2.95]** | 1.34 [0.92; 1.92] | 1.75 [0.96; 3.22] | 1.54 [0.97; 2.46] |
